# Patient access schemes in Asia-pacific markets: current experience and future potential

**DOI:** 10.1186/s40545-014-0019-x

**Published:** 2015-02-16

**Authors:** Christine Y Lu, Caitlin Lupton, Shana Rakowsky, Zaheer-Ud-Din Babar, Dennis Ross-Degnan, Anita K Wagner

**Affiliations:** Department of Population Medicine, Harvard Medical School and Harvard Pilgrim Health Care Institute, Boston, USA; Jefferson Medical School, Philadelphia, USA; School of Pharmacy, University of Auckland, Auckland, New Zealand

**Keywords:** Patient access schemes, Risk sharing, Managed entry, Conditional coverage, Health technology assessment, High cost medicines, Access to medicines

## Abstract

**Objectives:**

Patient access (or risk-sharing) schemes are alternative market access agreements between healthcare payers and medical product manufacturers for conditional coverage of promising health technologies. This study aims to identify and characterize patient access schemes to date in the Asia-Pacific region.

**Methods:**

We reviewed the literature on patient access schemes over the last two decades using publicly available databases, Internet, and grey literature searches. We extracted key features of each scheme identified, including the drug, clinical indication, stakeholders involved, and details of the scheme. We categorized schemes according to a previously published taxonomy of scheme types and by country.

**Results:**

We identified 3 schemes in South Korea, 5 in New Zealand, and 98 in Australia. Most (97.2%; n = 103) schemes focused on pharmaceuticals, few on medical technologies. More than half of the schemes related to treatments for cancer and inflammatory diseases such as rheumatoid arthritis. The majority (77.4%; n =82) involved pricing arrangements. Evidence generation schemes were rarely used. About half (41.8%; n = 41) of schemes in Australia were hybrid by nature, consisting of pricing arrangements with a conditional treatment continuation component.

**Conclusions:**

Australia has the most experience with patient access schemes and its experience may provide useful insights for other Asia-Pacific countries. The main targets are pharmaceuticals likely to have high budget impact (due to high per-patient costs and/or large volumes of use), and pharmaceuticals that may be adopted more widely than indicated. With the proliferation of high-cost medicines, the use of schemes may increase to address rising cost pressures, consumer demands, and uncertainties, while attempting to provide patient access to innovative care within finite budgets. Future research is warranted to evaluate the performance of patient access schemes.

## Introduction

Many countries and insurance schemes are pursuing universal health coverage with the goal of ensuring that all people have access to needed health services without suffering financial hardship [1,2]. However, healthcare systems grapple with the challenges of funding clinically beneficial health services and medicines while ensuring that financing systems are sustainable.

In an effort to improve the efficiency of rising healthcare expenditures, many systems evaluate whether expected additional health benefits of a new technology justify its additional cost compared to existing treatments (cost-effectiveness or ‘value-for-money’) [3-5]. Healthcare systems in the Asia-Pacific region that require proof of value-for-money in coverage of medical technologies include Australia, New Zealand, Thailand, South Korea, and Taiwan [6-10]. Even in systems that do not explicitly consider cost, there is often a focus on the magnitude of health benefits, which are informally weighed against cost. Traditionally, coverage decisions on medical technologies such as medicines and devices have been binary in nature; based on evidence available at launch and the price set by the manufacturer, payers decide whether or not to reimburse a product. In recent years, various types of conditional coverage decisions have emerged. Patient access schemes (also known as managed entry schemes or risk-sharing agreements) are alternative market access agreements, typically between payers and manufacturers, to enable provisional or conditional coverage of promising health technologies [4,5,11]. There are three broad categories of schemes [5]. First, *outcome-based schemes* (also known as performance-based or effectiveness guarantee schemes) in which the price, level, or nature of reimbursement are tied to clinical or intermediate endpoints measured in the future and ultimately related to patients’ quality or quantity of life. Second, *evidence generation schemes* in which a positive coverage decision is conditioned upon the collection of additional evidence through clinical studies, which might result in continued, expanded, or withdrawn coverage. Third, *financially-based schemes* negotiate company contributions to the cost of a product (e.g., discounts or rebates, price-volume agreements, utilization caps) for a particular patient or population without linking reimbursement to health outcomes.

Interest in patient access schemes is growing due to increasing cost pressures, the need to balance the interests of patients, clinicians, manufacturers, and other stakeholders, and uncertainties due to incomplete information at the time that decisions must be made. The necessary evidence to demonstrate incremental effectiveness and cost-effectiveness of a new technology is often not available at the time of launch [12]. Therefore, uncertainties exist about whether the technology will deliver the promised health gains in routine clinical practice. Clinical and cost-effectiveness uncertainties result in budget uncertainties [12]. In addition, costs of innovative products may bankrupt households and threaten sustainability of systems. Patient access schemes offer an important option for systems to allow (some) patients (early) access to promising technologies that systems may not otherwise fund. At the same time, these schemes may reduce the financial risks that payers face in making decisions based on limited evidence, and in some cases, they facilitate collection of more evidence to support future decisions [4,5,11].

Little is known about experiences with patient access schemes in Asia-Pacific countries. The purpose of this study was to identify and categorize patient access schemes in this region. The results of this study will enhance understanding of the roles of patient access schemes in emerging Asia-Pacific markets and inform policy developments for enabling patient access to promising technologies.

## Methods

We reviewed the literature on patient access schemes. We searched in PubMed and Google Scholar for English-language articles published through July 2012 using the following terms; ‘conditional coverage’ , ‘conditional reimbursement’ , ‘managed entry’ , ‘risk sharing’ , ‘risk sharing agreement’ , ‘risk sharing, pharmaceuticals’ , ‘risk sharing scheme’ , ‘coverage with evidence’ , ‘value-based pricing, pharmaceuticals’ , ‘patient access scheme’ , ‘pay back schemes’ , ‘performance-based, pharmaceuticals’ , ‘outcome-based reimbursement, pharmaceuticals’ , ‘reimbursement mechanisms, pharmaceuticals’ , and ‘outcome guarantee’. We scanned titles and abstracts to select relevant articles to review. The reference lists of relevant articles were also reviewed for studies fitting our criteria that our search strategy may have missed. We also added relevant unpublished or ‘grey literature’ (reports, conference presentations, payer websites) that were identified during our search.

We extracted key features of the schemes: the technology, disease area, country, payer, manufacturer, scheme type, and agreement details (when reported). We categorized the schemes according to a published taxonomy [5] of scheme types: (1) outcome-based schemes, (2) evidence generation schemes, and (3) financially-based schemes. These scheme types have generally been termed “risk sharing schemes” or “patient access schemes” by payers or pharmaceutical companies. Consistent with the literature [13], we classified schemes with a “conditional treatment continuation” component that limits continued subsidy of medicines to patients who demonstrate a pre-specified adequate clinical response as outcome-based schemes. We deliberately included financially-based schemes, in contrast to previous reviews of patient access schemes that focused on outcome-based schemes only [13]. Although financially-based schemes do not directly address uncertainties in clinical outcomes, they can address uncertainties in cost-effectiveness and/or budget impact estimates. Their use is increasing rapidly because they may be more feasible than outcome-based schemes that require more information and are administratively burdensome. We did not limit our search to any country. We report on schemes identified in Asia-Pacific markets.

## Results

### Search results

Using the search terms in PubMed and Google Scholar, we found 2229 articles published between 1998 and July 2012. Reviewing article titles and abstracts, we excluded commentaries and articles that did not describe specific patient access schemes. Next, we reviewed the references of remaining articles and grey literature sources for additional examples of patient access schemes. Our search identified 299 schemes described in 146 publicly available sources (92 scientific articles, 18 electronic articles, 14 reports, 9 websites, and 13 conference presentations). From these we selected the 106 examples from the Asia-Pacific region.

### Participating countries

We identified 106 patient access schemes in 3 countries from the Asia-Pacific region (Table 1). Public payers were involved in all schemes. Most (92.5%; n = 98) have taken place in Australia (Table 2) [14-21]. The Australian Pharmaceutical Benefits Advisory Committee (PBAC) is an expert committee that reviews incremental effectiveness and cost-effectiveness of medicines for coverage under the Pharmaceutical Benefits Scheme. The PBAC has formally assessed evidence of cost-effectiveness since 1993 [4]. The Medical Benefits Advisory Committee is an equivalent committee that evaluates medical devices for coverage under the Medical Benefits Scheme.

**Table 1:** **Patient access schemes in the Asia-Pacific region by country, type and condition**

	**Australia**	**South Korea**	**New Zealand**	**Total**
Types				
Outcome-based	21	-	-	21
Evidence generation	3	-	-	3
Financially-based	33	3	5	41
Hybrid*	41	-	-	41
Conditions				
Cancer	29	-	2	31
Inflammatory Conditions	28	-	1	29
Infectious Disease	7	-	-	7
Pulmonary Hypertension	7	1	1	9
Other	27	2	1	30
Technology				
Pharmaceuticals	95	3	5	103
Medical devices	3	-	-	3
Subtotal	98	3	5	106

*Hybrid schemes involved both pricing arrangements and conditional treatment continuation

**Table 2:** **Identified patient access schemes in Australia**

**Technology**	**Indication**	**Company**	**Payer**	**Type**	**Source**
**Cancer (n = 29)**					
Dasatinib	CML	Bristol-Myers Squibb Australia Pty Ltd	PBS	Outcome-based	Medicare Australia [14]
Nilotinib	CML	Novartis Pharmaceuticals Australia Pty Ltd	PBS	Outcome-based	Medicare Australia [14]
Imatinib	GIST, adjuvant	Novartis Pharmaceuticals Australia Pty Ltd	PBS	Outcome-based	Medicare Australia [14]
Imatinib	GIST, metastatic	Novartis Pharmaceuticals Australia Pty Ltd	PBS	Outcome-based	Medicare Australia [14]
Lapatinib	Late stage metastatic breast cancer	GlaxoSmithKline Australia Pty Ltd	PBS	Outcome-based	Medicare Australia [14]
Imatinib	Rare cancers (eg dermatofibrosarcoma protuberans, chronic eosinophilic leukemia)	Novartis Pharmaceuticals Australia Pty Ltd	PBS	Outcome-based	Medicare Australia [14]
Pazopanib	RCC	GlaxoSmithKline Australia Pty Ltd	PBS	Financially-based; Outcome-based	Public Summary Document [18]
Bevacizumab	Metastatic colorectal cancerr	Roche Products Pty Ltd	PBS	Financially-based; Outcome-based	Public Summary Document [18]
Sorafenib	Advanced hepatocellular carcinoma	Bayer Australia Ltd	PBS	Financially-based; Outcome-based	Public Summary Document [18]
PET	Staging of newly diagnosed NSCLC, esophageal cancer, cancer of gastro-esophageal junction, head and neck cancer, suspected residual/metastatic/recurrent colorectal cancer, melanoma, head and neck cancer, ovarian cancer	Not specified	MBS	Evidence generation	Stafinski et al. 2010 [15]
Exemestane	Oestrogen-receptor positive advanced breast cancer in post-menopausal women	Pfizer Australia Pty Ltd	PBS	Financially-based	Public Summary Document [18]
Imiquimod	Superficial basal cell carcinoma	3 M Pharmaceuticals Pty Ltd	PBS	Financially-based	Public Summary Document [18]
Letrozole	Advanced breast cancer in postmenopausal women	Novartis Pharmaceuticals Australia Pty Ltd	PBS	Financially-based	Public Summary Document [18]
Docetaxel	SCCHN	Sanofi-Aventis Australia Pty Ltd	PBS	Financially-based	Public Summary Document [18]
Sunitinib	RCC	Pfizer Australia Pty Ltd	PBS	Financially-based	PBS Schedule [16], Public Summary Document [18]
Cetuximab	K-RAS wild mCRC	Merck Serono Australia Pty Ltd	PBS	Financially-based	Public Summary Document [18]
Vemurafenib	Untreated unresectable stage IIIC or stage IV melanoma	Roche Products Pty Ltd	PBS	Financially-based	Public Summary Document [18]
Imatinib	ALL	Novartis Pharmaceuticals Australia Pty Ltd	PBS	Financially-based; Outcome-based	PBPA Relativity Sheets [17], Public Summary Document [18], Medicare Australia [14]
Dasatinib	ALL	Bristol-Myers Squibb Australia Pty Ltd	PBS	Financially-based; Outcome-based	PBPA Relativity Sheets [17], Public Summary Document [18], Medicare Australia [14]
Azacitidine	CML	Celgene Pty Ltd	PBS	Financially-based; Outcome-based	PBS Schedule [16], Medicare Australia [14]
Azacitidine	AML	Celgene Pty Ltd	PBS	Financially-based; Outcome-based	PBS Schedule [16], Medicare Australia [14]
Azacitidine	Myelodysplastic syndrome	Celgene Pty Ltd	PBS	Financially-based; Outcome-based	PBS Schedule [16], Medicare Australia [14]
Imatinib	CML	Novartis Pharmaceuticals Australia Pty Ltd	PBS	Financially-based; Outcome-based	PBPA Relativity Sheets [17], Medicare Australia [14]
Trastuzumab	HER2+ early breast cancer	Roche Products Pty Ltd	PBS	Financially-based; Outcome-based	PBPA Relativity Sheets [17], Medicare Australia [14]
Sunitinib	GIST	Pfizer Australia Pty Ltd	PBS	Financially-based; Outcome-based	PBS Schedule [16], Medicare Australia [14]
Lenalidomide	Myelodysplastic syndrome	Celgene Pty Ltd	PBS	Financially-based; Outcome-based	PBS Schedule [16], Medicare Australia [14]
Lenalidomide	Multiple myeloma	Celgene Pty Ltd	PBS	Financially-based; Outcome-based	PBS Schedule [16], Medicare Australia [14]
Bortezomib	Multiple myeloma	Janssen-Cilag Pty Ltd	PBS	Financially-based, Outcome-based	PBS Schedule [16], PBPA Relativity Sheets [17], Public Summary Document [18], Medicare Australia [14]
Erlotinib	Non-small cell lung cancer	Roche Products Pty Ltd	PBS	Financially-based; Outcome-based	PBS Schedule [16], Medicare Australia [14]
**Inflammatory Conditions (n = 28)**					
Adalimumab	Ankylosing spondylitis	AbbVie Pty Ltd	PBS	Outcome-based	Medicare Australia [14]
Etanercept	Ankylosing spondylitis	Pfizer Australia Pty Ltd	PBS	Outcome-based	Medicare Australia [14]
Golimumab	Ankylosing spondylitis	Janssen-Cilag Pty Ltd	PBS	Outcome-based	Medicare Australia [14]
Infliximab	Ankylosing spondylitis	Janssen-Cilag Pty Ltd	PBS	Outcome-based	Medicare Australia [14]
Adalimumab	Crohn’s disease	AbbVie Pty Ltd	PBS	Outcome-based	Medicare Australia [14]
Infliximab	Complex refractory fistulising Crohn’s disease	Janssen-Cilag Pty Ltd	PBS	Outcome-based	Medicare Australia [14]
Adalimumab	Juvenile arthritis	AbbVie Pty Ltd	PBS	Outcome-based	Medicare Australia [14]
Etanercept	Juvenile arthritis	Pfizer Australia Pty Ltd	PBS	Outcome-based	Medicare Australia [14]
Adalimumab	Psoriatic arthritis	AbbVie Pty Ltd	PBS	Outcome-based	Medicare Australia [14]
Golimumab	Psoriatic arthritis	Janssen-Cilag Pty Ltd(	PBS	Outcome-based	Medicare Australia [14]
Infliximab	Psoriatic arthritis	Janssen-Cilag Pty Ltd	PBS	Outcome-based	Medicare Australia [14]
Pimecrolimus	Atopic dermatitis who are over 18 years of age	Novartis Pharmaceuticals Australia	PBS	Financially-based	Public Summary Document [18]
Infliximab	Crohn’s disease	Janssen-Cilag Pty Ltd	PBS	Financially-based; Outcome-based	PBPA Relativity Sheets [17], Medicare Australia [14]
Adalimumab	Complex refractory fistulising Crohn’s disease	AbbVie Pty Ltd	PBS	Financially-based; Outcome-based	PBPA Relativity Sheets [17], Medicare Australia [14]
Tocilizumab	Juvenile arthritis	Roche Products Pty Ltd	PBS	Financially-based; Outcome-based	PBPA Relativity Sheets [17], Medicare Australia [14]
Abatacept	RA	Novartis Pharmaceuticals Australia Pty Ltd	PBS	Financially-based; Outcome-based	PBS Schedule [16], Medicare Australia [14]
Infliximab	RA	Janssen-Cilag Pty Ltd	PBS	Financially-based; Outcome-based	PBS Schedule [16], Lu et al. 2007 [33], Pugatch et al. 2010 [21], Medicare Australia [14]
Rituximab	RA	Roche Products Pty Ltd	PBS	Financially-based; Outcome-based	PBS Schedule [16], Lu et al. 2007 [33], Pugatch et al. 2010 [21], Medicare Australia [14]
Tocilizumab	RA	Roche Products Pty Ltd	PBS	Financially-based; Outcome-based	PBS Schedule [16], Medicare Australia [14]
Certolizumab Pegol	RA	UCB Australia Proprietary Ltd	PBS	Financially-based; Outcome-based	PBS Schedule [16], Medicare Australia [14]
Adalimumab	RA	AbbVie Pty Ltd	PBS	Financially-based; Outcome-based	PBS Schedule [16], Lu et al. 2007 [33], Pugatch et al. 2010 [21], Medicare Australia [14]
Golimumab	RA	Janssen-Cilag Pty Ltd	PBS	Financially-based; Outcome-based	PBS Schedule [16], Medicare Australia [14]
Etanercept	RA	Pfizer Australia Pty Ltd	PBS	Financially-based; Outcome-based	PBS Schedule [16], Lu et al. 2007 [33], Pugatch et al. 2010 [21], Medicare Australia [14]
Adalimumab	Severe chronic plaque psoriasis	AbbVie Pty Ltd	PBS	Financially-based; Outcome-based	PBS Schedule [16], Lu et al. 2007 [33], Pugatch et al. 2010 [21], Medicare Australia [14]
Etanercept	Severe chronic plaque psoriasis	Pfizer Australia Pty Ltd	PBS	Financially-based; Outcome-based	PBS Schedule [16], Medicare Australia [14]
Infliximab	Severe chronic plaque psoriasis	Janssen-Cilag Pty Ltd	PBS	Financially-based; Outcome-based	PBS Schedule [16], Medicare Australia [14]
Ustekinumab	Severe chronic plaque psoriasis	Janssen-Cilag Pty Ltd	PBS	Financially-based; Outcome-based	PBS Schedule [16], Medicare Australia [14]
Etanercept	Severe chronic plaque psoriasis under 18	Pfizer Australia Pty Ltd	PBS	Financially-based; Outcome-based	PBS Schedule [16], Medicare Australia [14]
**Infectious Diseases (n = 7)**					
Abacavir	HIV infection	ViiV Healthcare Pty Ltd	PBS	Financially-based	Robertson et al. 2009 [19], Adamski et al. 2010 [20]
Tipranavir	HIV infection	Boehringer Ingelheim Pty Ltd	PBS	Financially-based	PBS Schedule [16], Public Summary Document [18]
Entecavir	Chronic hepatitis B in adults 16 years and older with evidence of active liver inflammation	Bristol-Myers Squibb Australia Pty Ltd	PBS	Financially-based	Public Summary Document [18]
Boceprevir	Chronic genotype 1 hepatitis C infection	Merck Sharp & Dohme (Australia) Pty Ltd	PBS	Financially-based	Public Summary Document [18]
Telaprevir	Chronic genotype 1 hepatitis C	Janssen-Cilag Pty Ltd	PBS	Financially-based	Public Summary Document [18]
Posaconazole	Invasive fungal infections, not responsive to or intolerant of, alternative therapy	Schering-Plough Pty Ltd	PBS	Financially-based	Public Summary Document [18]
Posaconazole	Prophylaxis of invasive fungal infections among high risk patients	Schering-Plough Pty Ltd	PBS	Financially-based	Public Summary Document [18]
**Pulmonary Hypertension (n = 7)**					
Sildenafil	PPH or PAH	Pfizer Australia Pty Ltd	PBS	Outcome-based	PBS Schedule [16], Medicare Australia [14]
Tadalafil	PPH or PAH	Eli Lilly Australia Pty Ltd	PBS	Outcome-based	PBS Schedule [16], Medicare Australia [14]
Treprostinil Sodium	PPH or PAH	Orphan Australia Pty Ltd	PBS	Financially-based	Public Summary Document [18]
Ambrisentan	PPH or PAH	GlaxoSmithKline Australia Pty Ltd	PBS	Financially-based; Outcome-based	PBS Schedule [16], Medicare Australia [14]
Bosentan	PPH or PAH	Actelion Pharmaceuticals Australia Pty Ltd	PBS	Financially-based; Outcome-based	PBS Schedule [16], PBPA Relativity Sheets [17], Public Summary Document [18], Medicare Australia [14]
Epoprostenol	PPH or PAH	GlaxoSmithKline Australia Pty Ltd	PBS	Financially-based; Outcome-based	PBS Schedule [16], PBPA Relativity Sheets [17], Public Summary Document [18], Medicare Australia [14]
Iloprost trometamol	PPH or PAH	Bayer Australia Ltd	PBS	Financially-based; Outcome-based	PBS Schedule [16], Medicare Australia [14]
**Other (n = 27)**					
Modafinil	Narcolepsy	bioCSL (Australia) Pty Ltd	PBS	Outcome-based	Medicare Australia [14]
Verteporfin	Age-related macular degeneration	Novartis Pharmaceuticals Australia Pty Ltd	PBS	Outcome-based	Medicare Australia [14]
Deep brain stimulation	Patients with Parkinson's disease no longer responsive to drug therapy	Not specified	MBS	Evidence generation	Stafinski et al. 2010 [15]
EVAR	Abdominal aortic aneurysm	Not specified	MBS	Evidence generation	Stafinski et al. 2010 [15]
Dabigatran	Prevention of stroke or systemic embolism in patients with non-valvular atrial fibrillation	Boehringer Ingelheim Pty Ltd	PBS	Financially-based	Public Summary Document [18]
Atomoxetine Hydrochloride	ADHD diagnosed between the ages of 6 and 18 years	Eli Lilly Australia Pty Ltd	PBS	Financially-based	Public Summary Document [18]
Fingolimod	RRMS	Novartis Pharmaceuticals Australia Pty Ltd	PBS	Financially-based; Outcome-based	Public Summary Document [18]
Cinacalcet Hydrochloride	End stage renal disease receiving dialysis who have uncontrolled secondary hyperparathyroidism	Amgen Australia Pty Ltd	PBS	Financially-based	PBS Schedule [16], Public Summary Document [18]
Clostridium botulinum type A toxin-haemagglutinin complex	Severe spasticity of the upper limb in adults following a stroke, as an adjunct to physical therapy	Ipsen Pty Ltd	PBS	Financially-based	Public Summary Document [18]
Fentanyl citrate	Fentanyl lozenges for the treatment of breakthrough pain	Orphan Australia Pty Ltd	PBS	Financially-based	Public Summary Document [18]
Natalizumab	RRMS	Biogen Idec Australia Pty Ltd	PBS	Financially-based	Public Summary Document [18]
Paliperidone	Schizophrenia	Janssen-Cilag Pty Ltd	PBS	Financially-based	Public Summary Document [18]
Botulinum toxin type a purified neurotoxin complex	Moderate to severe spasticity of the upper limb in adults following a stroke as an adjunct to physical therapy	Allergan Australia Pty Ltd	PBS	Financially-based	Public Summary Document [18]
Tenofovir Disoproxil Fumarate	Treatment of chronic hepatitis B	Gilead Sciences Pty Ltd	PBS	Financially-based	Public Summary Document [18]
Pramipexole hydrochloride	Idiopathic Parkinson disease	Boehringer Ingelheim Pty Ltd	PBS	Financially-based	Public Summary Document [18]
Tobramycin	Pseudomonas aeruginosa respiratory infection in a patient with Cystic Fibrosis	Novartis Pharmaceuticals Australia Pty Ltd	PBS	Financially-based	Public Summary Document [18]
Ticagrelor	ACS, MI, or unstable angina	AstraZeneca Pty Ltd	PBS	Financially-based	Public Summary Document [18]
Pregabalin	Neuropathic pain	Pfizer Australia Pty Ltd	PBS	Financially-based	Public Summary Document [18]
Rivaroxaban	Acute symptomatic DVT	Bayer Australia Ltd	PBS	Financially-based	Public Summary Document [18]
Aztreonam	Pseudomonas aeruginosa infection in patients with cystic fibrosis	Gilead Sciences Pty Ltd	PBS	Financially-based	Public Summary Document [18]
Crinone (progesterone gel)	Supplement progesterone in women who have luteal phase defect	Merck Serono	PBS	Financially-based	Robertson et al. 2009 [19], Adamski et al. 2010 [20]
Deferasirox (Exjade)	Chronic iron overload in patients with disorders of erythropoiesis	Novartis	PBS	Financially-based	Robertson et al. 2009 [19], Adamski et al. 2010 [20]
Omalizumab	Uncontrolled severe allergic asthma	Novartis Pharmaceuticals Australia Pty Ltd	PBS	Financially-based; Outcome-based	PBS Schedule [16], Medicare Australia [14]
Aflibercept	Age-related macular degeneration	Bayer Australia Ltd	PBS	Financially-based; Outcome-based	PBS Schedule [16], Medicare Australia [14]
Ranibizumab	Age-related macular degeneration	Novartis Pharmaceuticals Australia Pty Ltd	PBS	Financially-based; Outcome-based	PBS Schedule [16], PBPA Relativity Sheets [17], Public Summary Document [18], Medicare Australia [14]
Eltrombopag	Severe chronic immune idiopathic thrombocytopenic purpura	GlaxoSmithKline Australia Pty Ltd	PBS	Financially-based; Outcome-based	PBPA Relativity Sheets [17], Medicare Australia [14]
Romiplostim	Severe chronic immune idiopathic thrombocytopenic purpura	Amgen Australia Pty Ltd	PBS	Financially-based; Outcome-based	PBS Schedule [16], Medicare Australia [14]

ACS = Acute Coronary Syndromes; ALL = Acute Lymphoblastic Leukemia; AML = Acute Myeloid Leukemia; CML = Chronic Myeloid Leukemia; DVT = Deep Vein Thrombosis; EVAR = endovascular aneurysm repair; GIST = Gastrointestinal Stromal Tumor; MBS = Medicare Benefits Schedule; mCRC = Metastatic Colorectal Cancer; MI = Myocardial Infarction; PAH = Pulmonary Arterial Hypertension; PBS = Pharmaceutical Benefits Scheme; PPH = Primary Pulmonary Hypertension; RA = Rheumatoid Arthritis; RCC = Renal Cell Carcinoma; RRMS = Relapsing-Remitting Multiple Sclerosis; SCCHN = Squamous Cell Carcinoma of the Head and Neck.

Apart from Australia, we found 5 schemes in New Zealand and 3 in South Korea. Table 3 summarizes the schemes [22-25]. These countries also have established a formal process for health technology assessment. Similar to Australia, New Zealand has a national health insurance for its people. The pharmaceutical management agency was established in 1993 to make decisions about drug coverage. The agency considers a number of factors, including the clinical need, clinical benefits and risks, cost-effectiveness, and budget impact of new drugs [26]. South Korea is the first country in Asia to officially adopt economic evaluation for making drug reimbursement decisions since January 2008. The Health Insurance Review Agency is responsible for reviewing the cost-effectiveness and budget impacts of new drugs for reimbursement under South Korea’s National Health Insurance [27].

**Table 3:** **Identified patient access schemes in the Asian-Pacific region (excluding Australian examples)**

**Country**	**Drug**	**Indication**	**Company**	**Payer**	**Type**	**Details**	**Source**
South Korea	Migraine medications	Migraine headaches	Not specified	National health insurance	Financially-based	Pharmaceutical companies maintained their PLS status via voluntary price cuts	Lee et al. 2012 [22]
	Hypertension medications	Hypertension	Not specified	National health insurance	Financially-based	285 of the 1226 hypertension drugs instituted price reductions following reassessment of clinical usefulness & price. Drugs were delisted if they failed to show a level of clinical usefulness or if their prices were higher than 80 percentile of the highest price among drugs containing the same ingredients. If the company accepted price cuts, the price was to be lowered to the level of the 80 percentile within 3 years	Lee et al. 2012 [22]
	Hyperlipidemia medications - therapeutic class	Hyperlipidemia	Not specified	National health insurance	Financially-based	Pharmaceutical companies maintained their PLS status via voluntary price cuts	Lee et al. 2012 [22]
New Zealand	Beta-interferon products, glatiramer acetate	Multiple sclerosis	Bayer	PHARMAC	Financially-based	Extended coverage for a specified number of patients	Raftery 2008 [23]
	Trastuzumab	Breast cancer	Roche	PHARMAC	Financially-based	Established separate hospital program to fund distribution of cancer drugs	Raftery 2008 [23]
	Imatinib	Chronic myeloid leukemia	Novartis	PHARMAC	Financially-based	Offered overall price reduction	Raftery 2008 [23]
	Atorvastatin	Hypertension	Pfizer	PHARMAC	Financially-based	Price volume agreements; manufacturer committed to pay for the drug if the sales exceed a fixed threshold	Antonanzas et al. 2011 [24]
	Adalimumab	Arthritis	Abbott Laboratories NZ Ltd	PHARMAC	Financially-based	Adalimumab spending is probably overstated due to a risk sharing agreement between the sponsor and PHARMAC, which involves rebates paid by the sponsor once Government spending reaches a certain level	Access Economics report for Arthritis New Zealand 2010 [25]

HIRA = Health Insurance Review & Assessment Service; PLS = positive list system; PHARMAC = The Pharmaceutical Management Agency.

### Types of medical products

Almost all patient access schemes focused on pharmaceuticals (97.2%; n = 103), few on medical technologies. More than half of schemes covered treatments for non-communicable diseases, predominantly cancer (n = 31) and inflammatory diseases (n = 29) such as rheumatoid arthritis. Schemes in Australia were largely established for technologies with high budget impact due to high cost per patient (e.g., adalimumab, which costs approximately $20,000 per patient-year) or large volumes of use (e.g., dabigatran for prevention of stroke or systemic embolism), as well as for products that may be used beyond their approved indications (e.g., entecavir that is subsidized only for chronic hepatitis B in adults with evidence of active liver inflammation). Similarly, New Zealand targeted new products with high cost per-patient or large volumes of use. Instead of individual drugs, the schemes in South Korea targeted the entire class of older medications with high budget impact due to large volumes of use (e.g., hyperlipidemia medications).

### Outcome-based schemes

Nearly two-thirds of schemes in Australia (63.3% n = 62; Table 2) included a “conditional treatment continuation” component that limits continued subsidy of high-cost medicines to patients who demonstrate an adequate clinical response. These schemes specify strict criteria for both initial and continued access. For example, the scheme for adalimumab for rheumatoid arthritis requires confirmation of clinical improvement at 3 months (measured by reductions in the total number of affected joints and in levels of inflammation markers) for continued subsidy [28]. In the scheme for bosenten in Australia, price was directly linked to the observed survival of patients treated with bosentan to confirm the clinical benefits (i.e., survival) and cost-effectiveness assumed at the time of assessment [29].

### Evidence generation schemes

We found three schemes with an evidence generation component in Australia, all focused on medical technologies: positron emission tomography, deep brain stimulators, and endovascular aneurysm repair (Table 2). In all three cases, the Australian government agreed to provide interim funding for access to the technology and to support collection of relevant clinical (and cost) information [15]. The manufacturers did not share the financial costs to generate evidence.

### Financially-based schemes

Financially-based schemes involving price reductions, price-volume agreements, or utilization caps were the most common risk-sharing schemes across the Asia-Pacific region (77.4%; n = 82). We identified 3 in South Korea, 5 in New Zealand, and 74 schemes in Australia. Forty-one of 74 schemes in Australia were hybrid schemes that involved both pricing arrangements and conditional treatment continuation.

## Discussion

In attempts to provide access to promising technologies, many countries are assessing the potential applicability of patient access schemes for their markets; these schemes typically involve novel arrangements between payers and manufacturers. We reviewed the literature on patient access schemes in the last two decades, with a focus on experiences in the Asia-Pacific region. We found a few schemes from South Korean and New Zealand, and many more schemes in Australia. Though different in their development and implementation, these schemes have arisen in response to cost pressures, demands from key stakeholders, and the inherent uncertainties in the health value and estimated budget impact of medical products in real-world settings. Pharmaceuticals account for nearly all identified schemes. The preponderance of pharmaceutical schemes may reflect the different evidence profiles at product launch, the higher budget impact of pharmaceuticals versus devices, and/or the different intellectual property and patent environments. There do not appear to be guidelines on when patient access schemes are applied or which type of scheme is most appropriate in a given circumstance. However, our findings suggest that the main targets are pharmaceuticals likely to have high budget impact due to high per-patient acquisition costs and/or potential large volumes of use, and pharmaceuticals likely to be used beyond their approved indications.

In the Asia-Pacific region, Australia has the most experience with patient access schemes. This may reflect the negotiating power and extensive experience in technology assessment, due to the design of the regulatory, institutional, and policy structures for medicines benefit decisions in the Australian publicly-funded national healthcare system [4,6]. High-cost medicines for treatment of cancer and inflammatory diseases were the targets of more than half of the schemes in Australia. Typical outcome-based schemes, in which the price or level of reimbursement is tied to achieving intermediate or final clinical endpoints, have rarely been used in Australia, with bosentan being the only example. Implementing outcome-based schemes is complex: they require detailed longitudinal information on patient clinical status (e.g., disease severity and progression, comorbidities) [30]; they require substantial financial, human, and infrastructure resources for monitoring of patients and financial transactions related to treatments [31]; and they require a mechanism for adjusting price or level of reimbursement when explicit clinical endpoints are not reached. Instead, Australia limits continued subsidy of high-cost medicines to patients who demonstrate an adequate response, known as “conditional treatment continuation” policies, which are often coupled with pricing arrangements, that is, hybrid schemes. Initial access is also restricted to a small pool of patients largely based on disease severity and non-responsiveness to less expensive therapeutic alternatives. Criteria restricting both initial and continued access aim to maximize value-for-money and control costs. While some evidence suggests that such policies can cap spending [28,32], whether they achieve value-for-money or if patients who need the medicines actually gained access should be investigated. Further, strict initial and continued access criteria are ethically challenging as individual patients may just *missed* the arbitrary threshold for access [33,34].

Evidence generation schemes were also rarely used in the Asia-Pacific region, possibly due to the complexity in tracking patient outcomes and the additional costs and administrative burden involved to operate these schemes.

In contrast, we found that financially-based schemes were common, possibly due to fewer operational challenges. Price-volume agreements and utilization caps have been in place for more than a decade in Australia [19]. Typically, product prices are reduced if sales exceed pre-agreed volumes, or expenditures refunded by the manufacturer if government expenditures exceed a pre-agreed cap or threshold. Price-volume agreements can shift cost considerations from the payer to the manufacturer, which is important especially if there are concerns that (i) new medicines will be prescribed to a wider population than envisaged, (ii) the patients prescribed the drug will not always be those most likely to gain the greatest benefit [4,11,35], and/or (iii) the product does not result in the expected clinical benefits. Expectations are that manufacturers will target promotion in accordance with approved prescribing requirements. Details of patient access schemes (e.g., capped prices, negotiated volumes) are generally unavailable to the public [19].

Our study has several limitations. The review focused on the Asia-Pacific region; however, the investigators were limited to the English language literature; schemes that were described only in other languages have not been included. Given the sensitive nature of contracts in this field, it is likely information on schemes remains unpublished. Manufacturers will be reluctant to disclose details about patient access schemes if such information will be available to other countries that use external reference pricing. We thus are likely to have underestimated the total number of patient access schemes in Asia-Pacific countries. Nevertheless, this study provides insights about what types of products are commonly targets for patient access schemes and about recent experiences in the Asia-Pacific region that may inform the development of future schemes.

Although beyond the scope of this review, we noted that industry-sponsored patient assistance programs were implemented in several countries to improve access to medicines. For instance, the Glivec International Patient Assistance Program, implemented since 2001 in 81 low and middle-income countries (including China, India, Indonesia, Thailand, and Vietnam), has provided access to imatinib for patients with specific types of leukemia or gastrointestinal cancer [36]. The MUSANDA patient assistance program, implemented in the United Arab Emirates, supports access to ranibizumab for age-related macular degeneration. Although payers are usually not involved in industry-sponsored patient assistance programs, these programs can provide interim access to innovative medicines while payer-industry patient access schemes are under negotiation; they can complement payer-industry patient access schemes by providing treatments for individual patients who are unable to self-fund treatments and who have no third-party payer; or they can become a vehicle for implementing a payer-industry patient access scheme, as has happened for coverage of imatinib in Thailand [37].

Globally, forecasts estimate that spending on new biologic agents will outpace overall spending growth on medicines and represent about 20% of the estimated US$1.2 trillion total pharmaceutical market by 2017 [38]. The recent availability of sofosbuvir, a new, curative treatment for hepatitis C, a disease that is highly prevalent in many low and middle-income countries, highlights the need for innovation in medicines financing. Refined patient access schemes may constitute much-needed innovations in financing arrangements that provide equitable and affordable access for those who need the new medicines. Payers have an incentive to develop strategies that can help control costs while ensuring patient access to medical products that benefit health. Patient access schemes may also mitigate the negative impacts of uncertainties in cost-effectiveness and budget impact estimates, and shift payer and manufacturer focus to improving patient outcomes in real-world settings – the ultimate goal of medical care. Payers have different ranges of authority over pricing, access, and evidence generation; some are more limited in the types of coverage decisions or access schemes they can consider, which may explain some of the variation in use of schemes between countries. Manufacturers are likely to prefer patient access schemes over a denial of coverage or explicitly reduced pricing, in part because patient access schemes can keep the real reimbursement prices confidential allowing them to tier prices by markets without the threat of external reference pricing [5,13].

There is little evidence on whether patient access schemes achieve their intended goals; they are a relatively recent development and the details of such agreements are usually confidential. However, key stakeholders appear willing to at least discuss types of schemes that can enable patient access to needed medicines [4,31,33]. Future studies should generate evidence on whether patients who need the medicines actually receive and benefit from them; whether schemes make high quality care more affordable for households and systems; whether they provide incentives for manufacturers to continue to invest in products that meet unmet needs; and whether data collected as part of the schemes confirm estimated cost-effectiveness or long-term benefits. Such evidence about potential benefits and costs is needed to inform the adoption of patient access schemes globally. Other factors that may affect their adoption center around operational challenges such as administrative burden and difficulty in tracking patient outcomes in healthcare delivery and financing systems [4,5,13,28,30-34,39], as well as the transparency and perceived fairness of the complex decisions on the establishment of patient access schemes and criteria for access [33,34]. Research is needed to compare costs of administration and operation on the payer side and effects on pricing strategies globally on the pharmaceutical company side between patient access schemes and upfront discounts in drug prices.

## Conclusions

Patient access schemes offer an important option for healthcare systems to allow patient access to promising technologies that may not otherwise be funded. Our study adds to existing knowledge by identifying and characterizing published patient access schemes in the Asia-Pacific region. Financially-based access schemes are most common. Australia tends to couple conditional treatment continuation with financial arrangements to provide further assurance. The main targets of patient access schemes are pharmaceuticals likely to have high budget impact due to high per-patient costs and/or large volumes of use, and pharmaceuticals that may be adopted more widely than indicated. With the rapid proliferation of high-cost medicines, these schemes may increasingly be used to enable access to innovative care within finite budgets. Future research is needed to generate evidence about the effectiveness and economic, administrative, and company pricing policy consequences of patient access schemes.
